# Adaptation and validation of a computer-assisted language learning attitude questionnaire in a Vietnamese EFL context: A comparison between online and paper modes of administration

**DOI:** 10.1016/j.heliyon.2022.e09743

**Published:** 2022-06-16

**Authors:** Lan Anh Thuy Nguyen, Anita Habók

**Affiliations:** a Doctoral School of Education, University of Szeged, Hungary; b Institute of Education, University of Szeged, MTA-SZTE Digital Learning Technologies Research Group, Hungary

**Keywords:** Computer-assisted language learning, Validity, Rasch analysis, Attitude, Confirmatory factor analysis, Exploratory factor analysis

## Abstract

The article reports on the validation of a computer-assisted language learning (CALL) attitude questionnaire and discusses differences between online and paper modes of administration, drawing on a sample of 1,769 Vietnamese undergraduates. Exploratory and confirmatory factor analyses were conducted to explore and assess the factor structure of the CALL instrument and specify the equivalence between the two versions of the questionnaire. Rasch model analysis was used to evaluate the overall fit and construct uni-dimensionality of the instrument. The findings of the study suggested a six-factor structure for the adapted questionnaire as well as both reliability and validity in the Vietnamese context. No significant difference was found between the two modes of administration as regards the construct and item levels of the questionnaire, although the paper-version was superior to the online version according to results from the Rasch model analysis. Hence, the instrument can be used in online and paper modes to measure Vietnamese tertiary students’ attitudes to the integration of technology into language learning. The study finds that further research is called for if the two modes of administration of the questionnaire are used in other contexts for teaching English as a foreign language (EFL).

## 1 Introduction

Computer-assisted language learning (CALL), which is defined as the process by which students use computers to improve their language learning (Beatty, 2010), has for years been an important part of acquiring a second language. CALL can aid students in different learning tasks, support the knowledge-constructed classroom (Muir-Herzig, 2004), empower students to be responsible for their learning, and create more opportunities to practice the language (Almekhlafi, 2006; Nguyen and Habók, 2021, 2022). The history of CALL could be categorized in three ways: (1) behaviorist, (2) communicative, and (3) integrative (Ürün, 2015). At the outset, the behaviorist approach to CALL involved repetitive language drills for instructional purposes. A communicative pedagogy then replaced behaviorism, thus creating more opportunities for students to practice through digital devices. The changing needs in language education in the 21^st^ century paved the way for integrative CALL, allowing students to practice their language skills in authentic environments while improving their technological capabilities. With the sheer growth of information and communication technology (ICT), the term CALL has been extended beyond the computer to applications (“apps”) and digital devices (Tafazoli et al., 2018). It has even been proposed that a culture component be incorporated in CALL to become “Computer-assisted Languacuture Learning” (Abolghasseminits et al., 2013; Chun et al., 2016; Zhu et al., 2009).

In educational institutions around the world, CALL has been extensively used for different purposes in language education and has become a fundamental feature of language teaching methodology to achieve learning objectives (Lodhi et al., 2019) through multimedia with video, sound, graphics, and text. Different studies have examined factors that impact the integration of digital applications in the classroom (Aslan and Zhu, 2017; Chen, 2008; Guillén-Gámez and Mayorga-Fernández, 2020). Several such studies have attempted to explore organizational factors and personal traits (Van Braak, 2001), overt and covert deterrents (Al-Kahtani, 2004), first- and second-order barriers (Yang and Huang, 2008), third-order barriers (Tsai and Chai, 2012), extrinsic and intrinsic barriers (Chen et al., 2012), and internal and external factors in “low resource” and “high resource” settings (Abedalaziz et al., 2013; Albirini, 2006; Al-ruz and Khasawneh, 2011; Aslan and Zhu, 2017; Atai and Dashtestani, 2011; Ifinedo et al., 2020; Lodhi et al., 2019). The research on ICT integration in the classroom finds that “human agency” is a significant element in the acceptance and efficacy of CALL (Abolghasseminits et al., 2013), so students' attitudes to the use of technological tools should be considered one of the vital issues in the successful use of technology in language learning (Ma et al., 2005). It is a well-established fact that attitudes bear a strong relationship with students’ behavioral intentions and computer usage (Ajzen and Fishbein, 2000), with positive CALL attitudes definitely impacting EFL learning (Levy and Hubbard, 2005).

In Vietnam, the Ministry of Education and Training has mandated multiple projects for implementing technology in education. The current project is titled “Enhancing the application of information technology in management and support for teaching–learning activities: Research on enhancing the quality of education and training in the 2016–2020 period with a view to 2025.” This has aided teachers and students in integrating technology with a variety of fields, including English as a foreign language (EFL) education. CALL has thus been implemented in several study areas in Vietnamese universities.

However, research in EFL education and technology has mostly focused on teachers' attitudes to CALL, even though both teachers' and students' attitudes are pertinent because learning will only take place if their attitudes are congruent (McGrail, 2005). Surveying learners' CALL attitudes may reveal challenges and opportunities for the education system (Aryadoust et al., 2016), given that understanding students' attitudes facilitates the integration of technologies into learning. Available research on Vietnamese teachers’ attitude to integrating ICT into English language teaching (e.g., Truong and Qalati, 2020) finds few questionnaires that address the comparison of paper-based and online questionnaire validity; or questionnaires translated into Vietnamese. The current study seeks to fill the gap by validating an instrument designed for both paper and online modes to be used for Vietnamese EFL learners.

## 2 Attitudes to CALL and the development of the construct

In the literature, the “attitude” has been defined in a number of studies. According to one school of thought, attitude refers to affective aspects of an individual (Cherry, 2019). Attitude is formed by experiences, viewpoint, cognition, and affect that drive an individual's perception of computers and other technological devices, people, or circumstances (Fishbein and Ajzen, 1975). Although attitude is considered to be latent, it can be measured (Bem, 1970) through students' responses to a specific subject (Abun et al., 2019) and ranked from negative to positive (Fiske, 2010). Attitude to CALL refers to students' or teachers' emotions tied to the use of technology (Joyce and Kirakowski, 2013) in language learning, and this has been investigated in different educational contexts (Abolghasseminits et al., 2013; Lodhi et al., 2019).

Many researchers have focused on three components to show the attitudes of EFL learners to the integration of technology into language acquisition: (1) cognitive, which refers to knowledge, perceptions, or ideas tied to technology use; (2) affective, which relates to emotions, or evaluations tied to the integration of ICT into education; and (3) behavioral, which is the expression of the intention or actions associated with teaching technology (Agyei and Voogt, 2011; Matteson et al., 2016). However, different authors have contributed to the methodology of tracking attitudes to CALL by incorporating and developing various constructs of teacher's or learner's attitude to integrating technology into language education. Some other components have been developed as part of the construct of ICT attitude in different studies, such as enjoyment (Christensen and Knezek, 2009; Kisanga and Ireson, 2016; Teo, 2006), anxiety (Agyei and Voogt, 2011; Alothman et al., 2017; Christensen and Knezek, 2009), avoidance (Christensen and Knezek, 2009), negativity (Christensen and Knezek, 2009), productivity (Atman Uslu and Usluel, 2019; Yavuz, 2005), and internal and external factors of ICT attitudes (Nagy and Habók, 2018). Students' attitudes to CALL have tended to be positive (Abolghasseminits et al., 2013; Ahmed, 2015; Liu, 2009; Lodhi et al., 2019), becoming more so with greater integration of technology into education.

## 3 CALL attitude assessment in the language classroom: an insight from the past to the present

The various frameworks or models that have been developed to measure attitudes to CALL for decades fall into two groups. The first directly measures an individual's attitude to technology. Among these frameworks and models, the theory of reasoned action (TRA) is considered one of the foundational models (Fishbein and Ajzen, 1975) for explaining the behavior of an individual through their attitudes to technology and subject norms (social referents, such as teachers and family members) and the relationship between the various components. The TRA construct has been widely applied to human attitudes and behavior in multiple fields, including language education (Almekhlafi, 2006). Different models have been developed or extended from the TRA subscales, such as the theory of planned behavior (TPB) (Ajzen, 1985) and the technology acceptance model (TAM) (Davis, 1989). Unlike TRA, the TPB model does not have an action factor. Instead, it uses the perceived behavioral control factor to specify an individual behavior that is resolved for the purpose of implementing the behavior and the subject norm. Modified from TRA, TAM shows that an individual's technology usage behavior is predicted through perceived utility and ease of use, user attitudes to technology, plans, and prospective adoption behavior.

TAM has been validated, used, and adapted in various studies on language learner attitudes and behaviors related to technology in language education (Rafique et al., 2020; Tan, 2019). The tripartite model also serves as a useful theoretical framework for developing attitude measures (Rosenberg and Hovland, 1960). The model includes three measurable components noted in the previous definition of attitude: (1) affect, (2) behavior, and (3) cognition. According to the theory, attitudes are a combination of predisposing factors (such as age and gender), affect (feelings about the object), beliefs (viewpoint of the object), and behavior (action taken involving the object). The theory proposes that the explanatory power of attitudes arises from these three constructs and is also influenced by various antecedent variables. Some other theories/models have an indirect relationship to learners' attitudes, such as the unified theory of acceptance and use of technology (Venkatesh and Davis, 2000) and the technology readiness and acceptance model (Lin et al., 2005). Different instruments have been generated from these theories and models to analyze language learners' attitudes to technology. Researchers mainly measure learners’ attitudes to CALL through Likert-scale questionnaires that have been designed on the basis of these frameworks/models (Lodhi et al., 2019).

It should be noted that the three components of attitudes to CALL noted above (behavior, affect, and cognition) have been widely applied in different studies (Dara Tafazoli et al., 2019; Teo, 2008) and generally viewed as the classical structure of attitude to CALL. However, they are not universally accepted by researchers (Hogg and Vaughan, 2011). Thus, in many empirical studies, different authors have incorporated different constructs to attitudes to integrating ICT into language education, albeit they are still linked to one or more of the three basic components. The CALL attitude structure has typically been viewed as multidimensional. Kearney, Gallagher, and Tangney (2020) developed and validated a five-construct instrument to measure learners' attitude to English and technology usage in learning the language: (1) behavioral engagement, (2) confidence in technology, (3) confidence in English, (4) engagement in emotions, and (5) using technology for learning. Behavioral engagement refers to the participation of an individual in classroom activities, whereas emotional engagement refers to reactions to academic tasks. English confidence and technology confidence specified the viewpoint, capability, and beliefs of language learners as regards learning EFL and the in-class and out-of-class use of technology, respectively. The using technology for learning construct aims to evaluate learner's perceptions of the application of technology to facilitate their EFL acquisition and achievement. In the same vein, a three-component CALL attitude instrument (behavioral/affective/language skills) that seeks to measure EFL learner's attitudes in applying technology in learning EFL has been extensively developed and validated in the Iranian EFL context by Aryadoust et al. (2016). Of note, this instrument can also be applied in low-technology settings. Teo (2006) used an abridged version of a questionnaire on computer attitude elaborated by Knezek et al. (1998) to evaluate Singaporean students' attitudes to computer use. The author selected three factors with 20 items (computer significance, computer interest, and technophobia) from the original version with 65 items categorized into eight factors (the significance of computers, enjoyment, motivation, study habits, passion, ingenuity, computer phobia, and seclusion). The abridged version of the CALL instrument assessed students' attitudes to technology in terms of cognitive and affective components of attitude. In a study by Vandewaetere and Desmet (2009), the construct of attitude to CALL comprised four components: (1) CALL's efficiency, (2) “surplus value of CALL,” (3) teacher impacts, and (4) barriers to CALL. It was also possible to re-organize these four subscales into the classical structure of attitude with three components because the first two components are interrelated with cognitive and affective factors, whereas two latter dimensions can be seen as a behavioral component. Nagy and Habók (2018) and Habók and Nagy (2017) developed and validated an eight-factor questionnaire to evaluate students' ICT attitude in the Hungarian EFL context, which consisted of internal and external components. The three-component instrument with the classical dimensions underpins different factors. Additionally, the authors also extended and linked the basic elements with other issues or digital devices in the modern language classroom, such as mobile devices, curriculum, and language learning tasks. This study will adapt and validate the instrument developed by Nagy and Habók (2018) in the Vietnamese EFL context.

## 4 Overview of the study

The study seeks to adapt and validate the CALL attitude instrument (Nagy and Habók, 2018; Habók and Nagy, 2017) in the Vietnamese context in both online and paper versions and compares the validity of the instrument between the two modes of administration modes. The process involves multiple steps:

1) Translation and back translation of items by teachers and experts; modifying the final version to involve both technology and paper;
2) Distributing the final draft of the instrument to the participants;
3) Exploring the structure of the instrument in the Vietnamese context; and
4) Comparing the instrument constructs and items based on the online and paper data.

The following research questions were formed to address the research objectives:

*RQ1. What evidence is there for the reliability and validity of the ICT attitude questionnaire in the Vietnamese context?*
*RQ2. Is there equivalence in the construct of the instrument and the results based on paper and online modes of administration?*
*RQ3. Is there equivalence at the item level of the instrument with respect to the dual modes of administration?*

## 5 Methodology

The study was conducted as cross-sectional research that uses exploratory factor (EFA), confirmatory factor (CFA), and Rasch model analyses. Before the research was performed, the Institutional Review Board at the Doctoral School of Education had provided ethical approval for the research. The authors confirm that informed consent was obtained from all Vietnamese participants, teachers, and rectors of the universities.

### 5.1 Participants

The questionnaire was administered both electronically and with hard copy documents to EFL undergraduate students at ten universities in Vietnam. There were 1,769 participants (28% male, 72% female; 7.3% freshmen, 23.3% sophomores, 15.8% juniors, 53.5% seniors; M_age_ = 20.98, SD_age_ = 1.79), who completed both the online and paper questionnaires. The sample was then split into two groups: an online cohort and a paper cohort. The online cohort (N = 1002) comprised 1,002 students (23.8% male, 76.2% female; M_age_ = 20.16; SD_age_ = 1.86). The paper cohort (N = 767) comprised 767 students (33.6% male, 66.4% female; M_age_ = 22.07, SD_age_ = 0.89), who used pen and paper to complete the questionnaire. The participants who volunteered for the research had different majors, from English to math to physics, but EFL was a compulsory course for all the participants. According to Kline (2016), 10–20 participants per parameter, or a minimum of 210 participants, are adequate to test the model. Thus, the number of students in the two groups (N = 1002 and N = 767) was sufficient.

### 5.2 The instrument and procedure

The Vietnamese CALL questionnaire was adapted from a questionnaire on attitudes to ICT tools (Nagy and Habók, 2018; Habók and Nagy, 2017). This is a self-report measurement tool that examines the attitude of language students to technologies in language learning through internal and external factors. The original questionnaire contains 28 four-point Likert-scale items ranging from disagree to agree and categorized into four internal factors (affective ICT strategies, metacognitive strategies, personal significance of ICT, significance of mobile devices) and four external factors (curriculum-based limitation, task-centered strategies, use of ICT tools in learning, and motivating role of ICT).

The adapted version of the 28-item questionnaire from the previous study (Habók and Nagy, 2017) was translated into Vietnamese; then, the two versions were compared for similarities and differences. The translated questionnaire was modified and improved several times by several researchers, IT teachers, and EFL teachers to ensure that all the questions were clear and could easily be understood by the students. During the translation, the researchers paid particular attention to cultural adaptation and key terms (e.g., ICT and virtual learning) and made some adjustments so that they are understandable and contextually suitable. The translated questionnaire items were assessed carefully in terms of conceptual, semantic, experiential, and operational equivalences. Additionally, an English-language version of the questionnaire that had been back translated from the Vietnamese version was compared with the original to check that all the instructions and items on the two English-language versions of the questionnaire were consistent with each other. Then, the final version of the Vietnamese questionnaire was modified into an online Google form and a paper document. The online form was designed to be convenient for the undergraduates to respond to the questions. All the functions of the online system were also checked carefully before the questionnaire was administered. Paper copies of the questionnaire were also organized carefully to aid the respondents. The two types of questionnaire had the same number of questions and the same content to aid data comparison.

## 6 Data collection procedure

### 6.1 Pen-and-paper questionnaire

The paper questionnaire was administered to the students in their classrooms. The students were notified that it was part of a research study, and the aims and ethical considerations were explained. The data collectors emphasized that the students’ responses would be used purely for research purposes and would not be divulged to anyone. The students were thus encouraged to answer all questions truthfully. The participants were then given time to reply to the paper questionnaire before their responses were collected by the data collectors. The data were then coded in an Excel file.

### 6.2 Online questionnaire and google forms

The electronic questionnaire was administered to the students in their online classrooms. Following the same procedure used for the paper questionnaire, the data collector explained the purpose of the research and related ethical issues. The students then logged in to the questionnaire on Google forms and responded to the questions. After completing the form and sending the answers to the data collector, all the data were available on the website and ready for analysis.

## 7 Data analysis

### 7.1 Exploratory factor analysis

First, EFA was done to explore the construct of the questionnaire in a Vietnamese context. IBM SPSS Statistics 22.0 was used on all the data to explore the dimensions of the instrument for assessing attitude to CALL in a Vietnamese context. The Kaiser–Meyer Olkin (KMO) statistic and Bartlett's sphericity test were implemented to estimate the level of intercorrelation and appropriateness of the sampling. The KMO statistic was used to test whether the factor analysis was reliable and whether the data were sufficient for the factor analysis. KMO values range between 0 and 1, and the index should be higher than 0.5 (Choi et al., 2011; Hair et al., 2017). According to Kaiser (1974), if a KMO value is greater than 0.90, it is highly significant; if it is between 0.80 and 0.90, it is notable; between 0.70 and 0.80, it is above average; between 0.60 and 0.70, it is mediocre; and between 0.50 and 0.60, it is merely acceptable.

### 7.2 Confirmatory factor analysis

CFA was then done with structural equation modeling in IBM SPSS AMOS 22.0 with a sample of the participants used to assess the research model. Normally, model fit indices used to check model fitness are categorized by absolute, comparative, and parsimonious fit (Schumacker and Lomax, 2004), with at least one index for each type reported (Hair et al., 2017). It is also recommended that the chi-square (χ^2^) value, comparative fit index (CFI), standardized root mean square residual (SRMR), and root mean square error of approximation (RMSEA) must have minimum indices to confirm the model fit (Kline, 2011). In the current study, the chi-square value, CFI, SRMR, and RMSEA indices were used to analyze the model fit (values to be discussed later). The main absolute fit index is χ^2^, which tests the null hypothesis of the fitness of the model and demonstrates whether the model fits the data. Although a significant χ^2^ shows that the model does not reproduce the data, a non-significant χ^2^ marks a good fit. χ^2^ statistics higher than 0.05 confirm a good relationship between the model and the data (Barrett, 2007). Nonetheless, it should be noted that χ^2^ has been found to be influenced by sample size, with the value increasing if the quantity of observed variables becomes greater. Hence, RMSEA and SRMR will be considered when assessing whether the model is well fitted to the data because those indices do not depend on sample size. RMSEA value normally ranges from 0 to 1. 0.10 > RMSEA >0.08 marks a meager fit, 0.08 > RMSEA >0.05 represents an acceptable fit, and 0.05 > RMSEA reflects a good fit (Browne and Cudeck, 1992). As RMSEA is also used to evaluate model complexity, the value is taken as an indicator of a parsimonious fit (Teo et al., 2013). SRMR is the index that shows the error extent from the estimation, thus reflecting the accuracy of the model, with a suggested cutoff value of 0.08 (Hu and Bentler, 1999). In the category of comparative fit, CFI is widely used to determine if the research model is superior to the null model. CFI values rank from 0 to 1, and a good value that is higher than 0.90 is related to a good model (Bentler, 1990).

The convergent and discriminant validity of the measurement model is also checked to reinforce assessment of the validity of the adapted instrument. Convergent validity measures how much items interact with one another. Fornell and Larcker (1981) proposed that convergent validity should be based on (1) the internal consistency reliability (Cronbach's alpha), (2) the average variance extracted (AVE), and (3) the composite reliability (CR, McDonald's coefficient omega; Raykov, 1997). Cronbach's alpha values should ideally be greater than 0.60 (Gliner et al., 2017) or higher than 0.70 (Hair et al., 2006). As regards CR, the CR value should exceed 0.70 (Nunnally and Bernstein, 1994) or 0.60 (Awang, 2012), and the AVE value should be over 0.50 (Hair et al., 2006). However, Fornell and Larcker (1981) hold that if the CR values are above 0.60, they are still acceptable once the AVE values are below 0.50. Discriminant validity measures whether items correlate with each other in one construct more than other items in another construct. Discriminant validity is confirmed if the square root of the AVE of an individual construct is higher than the squared factor correlation between the same construct and other constructs (Barclay et al., 1995). Discriminant validity of the instrument can also be assessed through the heterotrait–monotrait ratio of correlations (HTMT) (Henseler et al., 2015), with discriminant validity confirmed if HTMT values are below 0.85 (Kline, 2016).

### 7.3 Rasch model analysis

The Rasch measurement model has been widely applied in multiple studies that use a Likert scale to measure unobservable variables (Hendriks et al., 2012; Ishar and Masodi, 2012). Although EFA and CFA apply to the construct of instruments, the Rasch analysis concentrates on the pattern of item responses and expresses the mutual relationship between an individual and an item. Rasch model analysis assesses the strengths and weaknesses of the instrument as well as the precision of the construct, both individually and systematically (Boone, 2016). The Rasch approach does not attempt to change the model to fit the data but works from the opposite direction, enabling the instrument to be finessed by rescoring or removing items (Hendriks et al., 2012). Moreover, because Rasch analysis also uses individual and item parameters to measure the score of an item and requires the data to fit the model, researchers can measure how well the items on the instrument reflect the latent traits (Andrich, 2004). The individual items are analyzed and assessed through fit statistics –item fit and person fit. Item fit uses an index that involves item functionality, whereas person fit refers to an index that specifies the responses of a participant. Infit items (which really measure the latent trait) are expected to be in the 0 to 1 range. In the study, Rasch model analysis is employed to test the item fit in the modes of administration with the ACER ConQuest program (Adams & August, 2010).

## 8 Results

### 8.1 Research question 1: what evidence is there for the reliability and validity of the questionnaire in the Vietnamese context?

#### 8.1.1 Exploratory factor analysis

**Figure 1:**
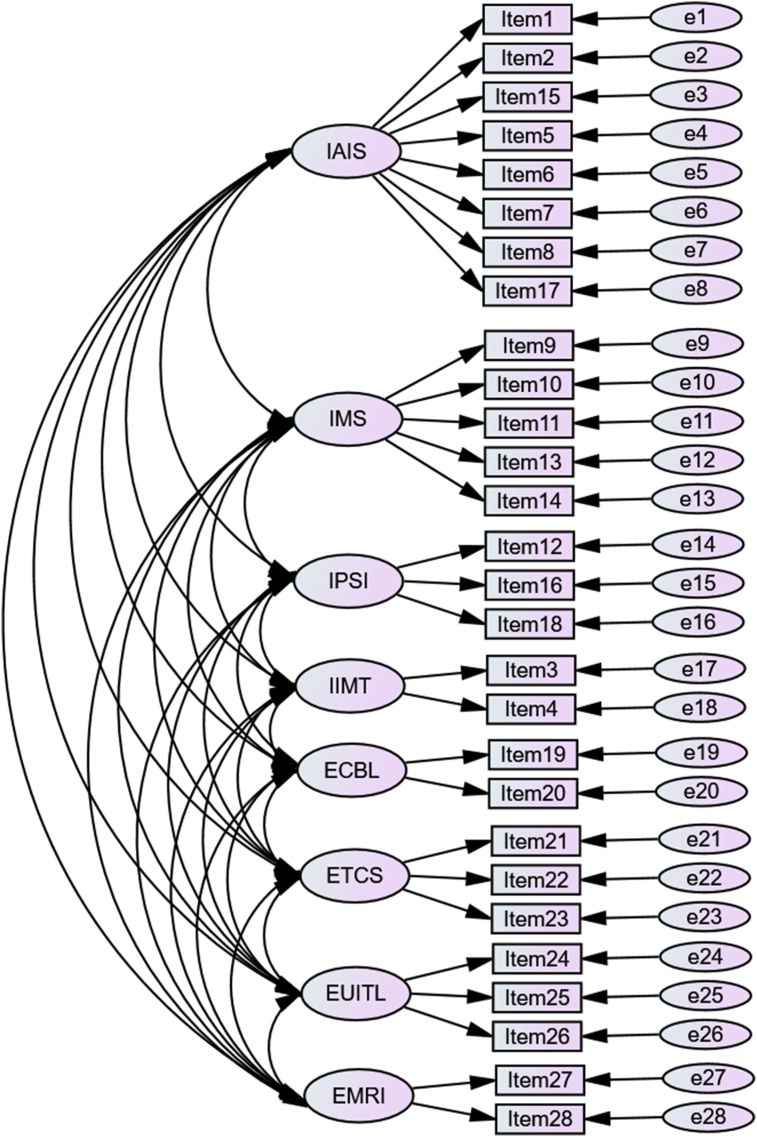
The eight-factor model (Habók and Nagy, 2017). *Note*: IAIS: internal affective ICT strategies, IMS: internal metacognitive strategies, IPSI: internal personal significance of ICT, IIMT: internal importance of mobile tools, ECBL: external curriculum-based limitation, ETCS: external task-centered strategies, EUITL: external use of ICT tools in learning, EMRI: external motivating role of ICT.

Analysis showed that the online and paper data collected from the students is appropriate for data analysis as the KMO index is 0.89 and Bartlett's sphericity is highly significant (p < 0.01). Although the original questionnaire contains four internal factors and four external ones with 28 items mentioned in the description of the instrument, the EFA suggested a six-factor model for the Vietnamese instrument with 27 items involving three internal factors and three external ones (Figure 1): (1) internal ICT importance (III –6 items), (2) internal affective attitude (IAA –6 items), (3) IMS (5 items), (4) external learning activities (ELA –3 items), (5) EUITL (4 items), and (6) External ICT facility and material limitation (EIFML –3 items). The labels for the six factors on the adapted Vietnamese questionnaire were adjusted to fit with the content of the items that fall within each factor; however, they were still named on the basis of the four internal factors and four external ones on the original questionnaire (Table 1). The six-factor structure of the instrument thus derived achieved a reasonable level of cumulative variance (53.174% in total) as values between 40% and 60% are adequate for social studies (Dunteman, 1989) with variance explained by each component described in Table 1.

**Table 1:** EFA for students’ attitudes to CALL (with varimax rotation).

Items	Statement	Factor loading
	(1) III (Eigenvalue = 7.405, Variance = 26.447%)	
**Item 9**	I can focus on English learning more if I use ICT tools.	0.509
**Item 10**	I can understand the English material much more easily if I use ICT tools.	0.681
**Item 11**	I can remember what I have learned better if I use ICT tools.	0.741
**Item 12**	ICT tools play an important role in my English learning process.	0.599
**Item 13**	ICT tools make English learning faster for me.	0.701
**Item 14**	ICT tools improve my English grades.	0.714
	(2) IAA (Eigenvalue = 2.328, Variance = 8.314%)	
**Item 4**	Using a tablet for English learning is very important to me.	0.419
**Item 5**	Using a computer for English learning makes me happy.	0.657
**Item 6**	Using ICT tools for English learning makes me happy.	0.661
**Item 7**	I use ICT tools for English learning because I am very interested in IT.	0.673
**Item 8**	I save time if I use a computer for English learning.	0.638
**Item 17**	I save time if I use ICT tools for English learning.	0.563
	(3) IMS (Eigenvalue = 1.654, Variance = 5.906%)	
**Item 2**	Using a computer for English learning is very important to me.	0.665
**Item 3**	Using a smartphone for English learning is very important to me.	0.703
**Item 15**	Using ICT tools for English learning is very important to me.	0.691
**Item 18**	Information is much more easily available by using ICT tools than by visiting the library.	0.534
**Item 28**	Teachers should incorporate the use of ICT tools into their English teaching.	0.494
	(4) ELA (Eigenvalue = 1.782, Variance = 6.363%)	
**Item 21**	Teachers give us guidance on how to use ICT tools for English learning tasks to be completed at home.	0.796
**Item 22**	Teachers give us guidance on how to use ICT tools for English learning in class.	0.869
**Item 23**	Teachers support the use of ICT tools for English learning.	0.841
	(5) EUITL (Eigenvalue = 1.415, Variance = 5.054%)	
**Item 1**	I use a computer as part of my English learning process.	0.634
**Item 24**	My teachers use a computer during their English classes.	0.586
**Item 25**	My teachers expect me to use a computer as part of my English learning process.	0.775
**Item 26**	Virtual English learning environments are used in the courses I am enrolled in.	0.712
	(6) EIFML (Eigenvalue = 1.090, Variance = 3.894%)	
**Item 16**	I cannot learn English without using ICT tools.	0.461
**Item 19**	The English material covered does not allow for the use of ICT tools in class.	0.836
**Item 20**	The English material covered does not allow for the use of ICT tools at home.	0.823

#### 8.1.2 Confirmatory factor analysis

**Figure 2:**
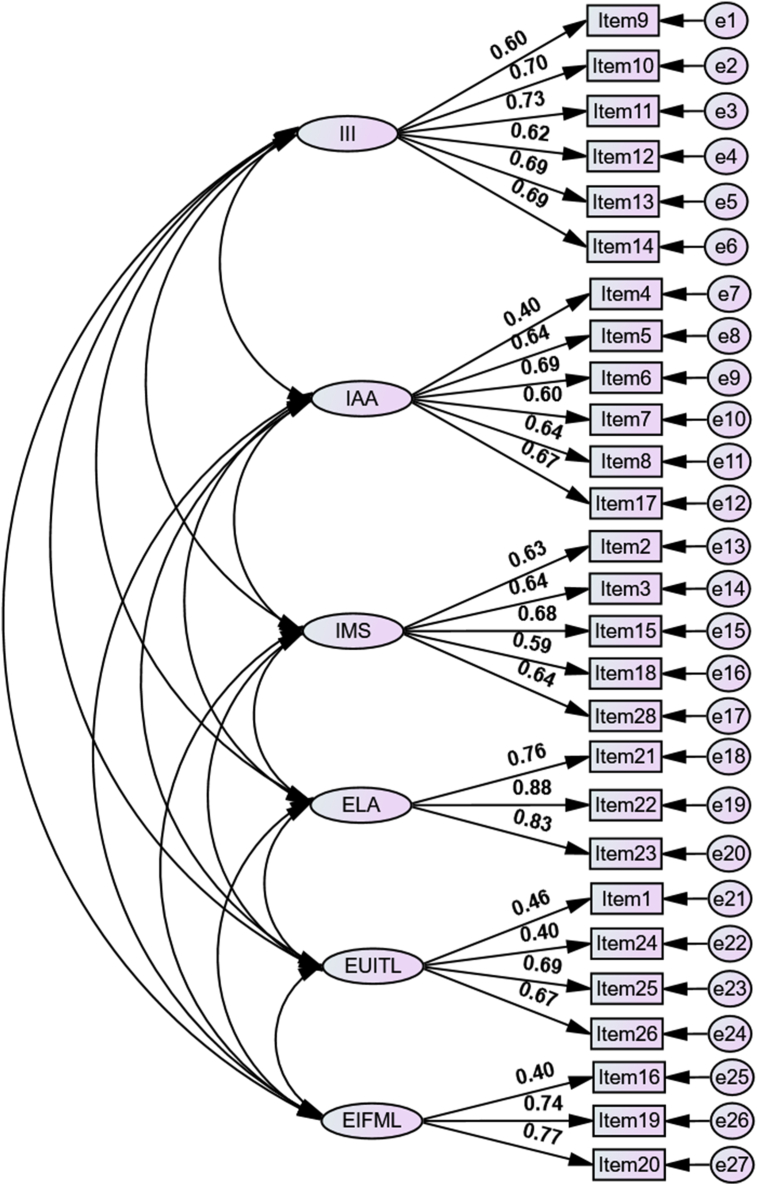
The six-factor structural model (N = 1769).

After the structure of the instrument was determined for the Vietnamese context, the six-factor solution was then evaluated on the basis of the model fit indices. The CFA of the model (Figure 2) showed acceptable indices (χ^2^ = 1560.940; df = 237; CFI = 0.901; RMSEA = 0.056; SRMR = 0.053). The model also demonstrated an acceptable level for convergent validity. The analysis showed that the questionnaire has high reliability (α = 0.88, ω = 0.90). The reliability of each factor was also supported by Cronbach's alpha for each factor, with ranks from 0.62 to 0.85 and with omega values in the 0.67–0.90 range. III and ELA showed a high level of reliability (α = 0.85, ω = 0.90; α = 0.87, ω = 0.87) with good Cronbach's alpha and omega for IAA and IMS (α = 0.79, ω = 0.88; α = 0.74, ω = 0.77). EUITL and EIFML had slightly lower values but were still acceptable (α = 0.62, ω = 0.69; α = 0.63, ω = 0.67). The model showed some AVE values for III, IAA, IMS, and EUITL as being lower than 0.50 (Table 2) but CR values for all factors being higher than 0.60; hence, the model was found to be valid in terms of convergence (Fornell and Larcker, 1981).

**Table 2:** Convergent validity measures.

Factors	Cronbach's α (>0.60)∗	ω (>0.60)∗	(AVE) (>0.50)∗	(CR) (>0.60)∗
1. III	0.85	0.90	0.43	0.82
2. IAA	0.79	0.88	0.37	0.77
3. IMS	0.74	0.77	0.38	0.75
4. ELA	0.87	0.87	0.69	0.87
5. EUITL	0.62	0.69	0.46	0.77
6. EIFML	0.63	0.67	0.53	0.76
Total	0.88	0.90	0.45	0.95

*Note*: ∗Sufficient level of reliability or validity; CR was calculated using (Σλ)2/(Σλ)2 + Σ(1 − λ2); AVE was calculated using Σλ2/Σλ2 + Σ(1 − λ2).

As regards the discriminant validity of the questionnaire, the square root of AVE and inter-construct correlation in the component correlation matrix was compared, confirming the discriminant validity of the model because the square root of AVE was above the inter-construct correlation values between the factors. The values are shown in Table 3.

**Table 3:** Discriminant validity measures.

Component correlation matrix						
Factors	III	IAA	ELA	IMS	EUITL	EIFML
**1. III**	.642∗					
**2. IAA**	.541	.583∗				
**3. IMS**	.292	.290	.616∗			
**4. ELA**	.432	.329	.423	.616∗		
**5. EUITL**	.147	.110	.040	−.080	.707∗	
**6. EIFML**	.278	.290	.251	.073	.061	.721∗

*Note*: ∗The square root of average variance extracted value.

#### 8.1.3 Rasch model analysis

Moreover, Rasch model analysis found that item and person fit statistics were acceptable for all the items on the questionnaire with deviance = 94123.071, p < 0.01 (Tables 6 and 7) and with values ranging from 0.85 to 1.42. For III and IAA, items 14 and 6 best fit in terms of person fit, respectively, whereas items 3 and 23 fit well in terms of person fit for IMS and ELA, respectively. For EUITL, the most appropriate item is item 1, while item 19 was the most suitable for EIFML.

### 8.2 Research question 2: is there equivalence in the construct of the instrument and the results based on the two modes of administration?

In an effort to address the second research question, which aims to examine the equivalence in the construct of the instrument based on the two modes of administration, data collected from the online (N = 1002) and paper-based (N = 769) versions of the instrument were separated and then tested using the six-factor model with the two types of data. Both types of data fit the model because all the values reached a good or acceptable level, although the CFI for the paper data is slightly lower than that of the online data (Table 4).

**Table 4:** Fitness indices of the six-factor model for the online and paper instruments.

Six-factor model	χ^2^	df	CFI (>0.90)∗	RMSEA (<0.08)∗	SRMR (<0.08)∗
Online	847.035	237	0.917	0.051	0.048
Paper	1039.166	237	0.870	0.066	0.062

*Note*: ∗Acceptable level of model fit indices.

Additionally, the online and paper instruments achieved a good level of reliability (α = 0.87, ω = 0.89; α = 0.90, ω = 0.91 respectively). The Cronbach's alpha and omega values for the two versions of the questionnaire had good or acceptable levels of reliability for each factor (see Table 5). The levels of reliability of the two types of questionnaire were consistent with each other. Although III and ELA attained the highest reliability, this value in IAA and IMS was slightly lower. EUITL and EIFML achieved the lowest level of reliability compared with the other factors but were still acceptable. As with the full sample (N = 1769), the AVE values are lower than 0.50 in some factors in both versions of the instrument. Nevertheless, the convergent validity of the electronic and paper instruments was still validated, as the CR values for all factors were above 0.60 (Fornell and Larcker, 1981) (see Table 5).

**Table 5:** Convergent validity of the online and paper questionnaires.

Factors	Cronbach's α (>.60)∗		ω (>.60)∗		AVE (>.50)∗		CR (>.60)∗	
	Online	Paper	Online	Paper	Online	Paper	Online	Paper
**1. III**	0.84	0.86	0.90	0.92	0.43	0.50	0.81	0.83
**2. IAA**	0.76	0.82	0.91	0.89	0.57	0.39	0.84	0.76
**3. IMS**	0.71	0.71	0.74	0.75	0.46	0.27	0.72	0.72
**4. ELA**	0.87	0.87	0.87	0.87	0.38	0.61	0.76	0.85
**5. EUITL**	0.70	0.47	0.75	0.56	0.52	0.74	0.81	0.85
**6. EIFML**	0.68	0.56	0.71	0.62	0.54	0.66	0.77	0.79
**Total**	0.87	0.89	0.90	0.91	0.47	0.46	0.95	0.95

Note: CR was computed using (Σλ)2/(Σλ)2 + Σ(1 − λ2); AVE was computed using Σλ2/Σλ2 + Σ(1 − λ2).

Moreover, the discriminant validity of the paper and online instruments was also confirmed, as the HTMT ratio values of both versions were lower than 0.85 for all six factors (Henseler et al., 2015). The discriminant validity values for the electronic and paper instruments are shown in Tables 6 and 7, respectively.

**Table 6:** HTMT ratios from the correlations between the components of the online instrument.

Factors	III	IAA	IMS	ELA	EUTIL	EIFML
1. III	1	0.575	0.489	0.721	0.078	0.694
2. IAA		1	0.615	0.631	0.033	0.650
3. IMS			1	0.701	0.102	0.406
4. ELA				1	0.025	0.489
5. EUTIL					1	0.025
6. EIFML						1

**Table 7:** HTMT ratios from the correlations between the components of the paper instrument.

Factors	III	IAA	IMS	ELA	EUITL	EIFML
1. III	1	0.788	0.704	0.459	0.211	0.241
2. IAA		1	0.790	0.432	0.297	0.300
3. IMS			1	0.523	0.172	0.208
4. ELA				1	0.227	0.159
5. EUITL					1	0.129
6. EIFML						1

Thus, the construct of the electronic and paper versions is equivalent because the convergent and discriminant validity of both types of instrument was confirmed.

### 8.3 Research question 3: is there equivalence at the item level of the instrument with respect to the dual modes of administration?

The six-factor model was specified through EFA and then confirmed through CFA, with a Rasch model analysis conducted on the subscale level. After comparing the construct of the electronic and paper instruments, item fit was analyzed with the partial credit model because the likelihood ratio test was significant (p < .001). The analysis showed that all individual items on both types of instrument have a good fit, with an infit and outfit ranking from 0.88 to 1.43 for the online data, whereas the values for the paper data fell within the 0.81–1.43 range. A summary of fit statistics for all the data (online and paper data) is shown in Table 8.

**Table 8:** Rasch model analysis of the items on the online and paper instruments.

Factors	Items	Online			Paper			Full sample		
		Location	SE	Weighted fit	Location	SE	Weighted fit	Location	SE	Weighted fit
**1. III**	Item 9	0.096	0.029	0.95	0.616	0.034	0.91	0.379	0.021	0.92
	Item 10	−0.083	0.030	0.93	0.039	0.035	0.82	−0.036	0.022	0.87
	Item 11	0.039	0.029	0.94	0.285	0.034	0.85	0.139	0.021	0.89
	Item 12	−0.265	0.031	0.89	−0.480	0.036	0.85	−0.347	0.023	0.87
	Item 13	−0.241	0.031	0.87	−0.363	0.036	0.83	−0.274	0.023	0.85
	Item 14	0.011	0.029	0.93	0.265	0.034	0.90	0.100	0.022	0.90
**2. IAA**	Item 4	0.179	0.029	1.09	0.395	0.034	1.05	0.252	0.021	1.09
	Item5	−0.180	0.031	0.97	−0.265	0.036	0.90	−0.213	0.023	0.93
	Item 6	−0.294	0.031	0.94	−0.332	0.036	0.80	−0.285	0.023	0.89
	Item 7	0.038	0.030	0.95	0.195	0.034	0.84	0.108	0.022	0.93
	Item 8	−0.138	0.031	0.87	0.047	0.034	0.90	−0.016	0.022	0.90
	Item 17	−0.204	0.031	0.87	−0.287	0.035	0.81	−0.245	0.023	0.86
**3. IMS**	Item 2	−0.343	0.033	1.00	−0.685	0.037	0.89	−0.515	0.024	1.00
	Item 3	−0.334	0.033	0.94	−0.905	0.038	0.92	−0.601	0.024	0.97
	Item 15	−0.309	0.033	0.97	−0.906	0.037	0.86	−0.548	0.024	0.94
	Item 18	−0.372	0.033	0.89	−0.657	0.037	0.99	−0.510	0.023	0.95
	Item 28	−0.252	0.032	0.87	−0.986	0.038	0.97	−0.557	0.023	0.95
**4. ELA**	Item 21	−0.008	0.031	0.88	−0.021	0.035	0.99	−0.022	0.022	0.93
	Item 22	−0.006	0.031	0.88	−0.227	0.035	0.97	−0.110	0.022	0.93
	Item 23	−0.025	0.031	0.84	−0.299	0.036	1.01	−0.161	0.023	0.93
**5. EUITL**	Item 1	0.229	0.028	1.46	0.550	0.035	1.08	0.352	0.021	1.29
	Item 24	−0.401	0.030	1.28	−0.695	0.036	1.23	−0.506	0.022	1.25
	Item 25	0.411	0.028	1.35	0.752	0.032	1.33	0.581	0.020	1.33
	Item 26	0.817	0.155	1.40	1.575	0.179	1.43	1.123	0.112	1.42
**6. EIFML**	Item 16	0.623	0.027	1.05	0.659	0.033	1.30	0.619	0.020	1.14
	Item 19	0.489	0.027	1.07	1.241	0.032	1.33	0.787	0.020	1.21
	Item 20	0.524	0.027	1.05	0.490	0.033	1.34	0.505	0.020	1.18

These findings suggest that all the individual items in each factor for both types of sample achieve good fit parameters and that all the items are suitable because they do not exceed the prescribed infinite range. Furthermore, a comparison of the deviance values for the two types of instruments (see Table 9) shows that, although all the items on both versions of the questionnaire fit very well in terms of person fit, the paper instrument is superior to the online one (deviance_paper_ = 39943.179, deviance_online_ = 46582.296).

**Table 9:** Model comparison for the online and paper questionnaires.

Model	Deviance	Parameters	p-value
Full sample	94123.071	82	<0.01
Online sample	46582.296		
Paper sample	39943.179		

## 9 Discussion and conclusions

This study provides a new structure of dimensionality for an instrument that assesses students' attitudes to CALL (Nagy and Habók, 2018; Habók and Nagy, 2017) in a Vietnamese EFL context. The study confirmed the validity of the adapted questionnaire and compared the validity of the instrument between online and paper modes at the construct and item levels. The questionnaire was translated into Vietnamese with due attention to technical terms and regional culture. Some adjustments were made to certain key terms on the questionnaire so that all the items would be appropriate for Vietnamese students and their knowledge. The final version of the adapted questionnaire was distributed to EFL learners electronically and physically. Both online and paper data were then used to validate the questionnaire. The collected online and paper data were initially used for EFA, which showed the structure of the CALL instrument in a Vietnamese EFL context with six components (which were different from those of the original version). The labels for these six factors were modified to fit with the items because some had been reconstructed in different factors: III (6 items), IAA (6 items), IMS (5 items), ELA (3 items), EUITL (4 items), and EIFML (3 items). As with the original instrument developed by Habók and Nagy (2017), although the instrument was structured differently, three basic elements (cognition, affect, and behavior) were reflected in these factors on the questionnaire. Six factors on the adapted questionnaire were re-organized on the basis of the three basic elements of attitude: cognitive (III, IMS, and EIFML), affective (IAA), and behavioral (EUITL and ELA). The current study takes the same approach as studies whose authors investigated all three basic elements or merely selected one out of three factors and linked them to other components to assess learners' attitudes to CALL. The factors were also grouped and renamed based on the fundamental structure of attitude (Teo, 2006; Vandewaetere and Desmet, 2009). However, Nagy and Habók (2018) and in the current study, the cognitive component of CALL attitude is broader than that of previous studies. It not only includes learners’ knowledge of the integration of technology into the language learning process but also their perception of materials or devices other than laptops in the modern classroom, such as tablets and smartphones. Although the six factors on the questionnaire were re-organized into the three classic elements, as noted above, the affective and cognitive components are not clear-cut. This has also been explored in previous research (e.g., Ajzen, 2005), and these two dimensions of attitude could be categorized in one component.

The values for the model fit indices show that the model fits with the data acceptably. The convergent and discriminant validity of the model was also demonstrated though the data analysis showed low AVE values on some sub-scales. The data were then subdivided into two groups based on the type of questionnaire, and these two types of data were used to test the fitness of the model and investigate the equivalence in the construct between the two versions of the instrument. Although the CFI value for the paper data is less than that of the online data, and a little lower than the suggested value (CFI _paper_ = 0.870), other values achieved an acceptable level. Thus, the online and paper data fit with the six-factor model. Moreover, Rasch model analysis further confirmed the structural validity of the six-factor model of the adapted Vietnamese CALL questionnaire, because all the items on the online and paper instruments fit well. At the item level, the paper version proved better than the online instrument because the deviance value of the former was less than that of the latter. Thus, the online and paper versions display no difference at the construct and item levels because the goodness of the construct level can complement the deficiency of the item level, and vice versa.

On the whole, the adapted, six-factor Vietnamese questionnaire is reliable and valid in a Vietnamese EFL context. Hence, it can be used either online or in the traditional pen-and-paper format. This study provides evidence for the reliability and validity of both the online and traditional paper-and-pen versions of the tool to assess EFL learners' attitude to the integration of technology into language education. This may benefit CALL research in Vietnam, given the paucity of validated instruments to assess learners’ attitude to CALL. Because the administration of the questionnaire in both modes attained satisfactory results, future research could adapt and use both versions of the instrument or use them interchangeably, especially in situations like the current pandemic period. Additionally, since the participants of the study are students in different years and come from a variety of majors, the questionnaire could be used or adapted in multiple EFL contexts in Vietnam to assess language learner attitudes to technology in foreign language education. Understanding the attitudes of learners, teachers, and other stakeholders could support the successful incorporation of educational technology into the language classroom.

Nevertheless, it is suggested that further investigation should be conducted if the two modes of administration of the questionnaire are used in other EFL contexts. This study only collected data from undergraduate students at certain Vietnamese universities; hence, the results cannot be generalized to the whole country or to other developing countries. Because the structure of Vietnamese students' attitude to CALL consists of internal and external dimensions, future research can examine the intercorrelation between components and other constructs. Furthermore, future research may explore the construct of the questionnaire in another context or the same context with more male students because female students were overrepresented in the current study. In addition, it is recommended that other researchers conduct studies that compare teachers' and students' attitudes in an EFL context because learning will not happen if their attitudes are not consistent (McGrail, 2005). Additionally, students' attitudes can change over time, so it is necessary to study language learners’ attitudes to CALL at regular intervals (Aryadoust et al., 2016) because of the shift from “low resource” to “high resource” settings and the significant growth of technologies. It has also been suggested that longitudinal studies can investigate the constancy of the model for the EFL context in Vietnam or other developing countries.

## Declarations

### Author contribution statement

Lan Anh Thuy Nguyen: Conceived and designed the experiments; Performed the experiments; Analyzed and interpreted the data; Contributed reagents, materials, analysis tools or data; Wrote the paper.

Anita Habók: Conceived and designed the experiments; Contributed reagents, materials, analysis tools or data; Wrote the paper.

### Funding statement

This work was supported by the Research Programme for Public Education Development, Hungarian Academy of Sciences (grant KOZOKT2021-16) and by the University of Szeged Open Access Fund (grant number: 5620).

### Data availability statement

The data that has been used is confidential.

### Declaration of interests statement

The authors declare no conflict of interest.

### Additional information

No additional information is available for this paper.

## Appendix A Supplementary data

Questionnaire_English R1 2022 01 08 Questionnaire_English R1 2022 01 08
