# Supplementary material for: Adaptation and validation of a computer-assisted language learning attitude questionnaire in a Vietnamese EFL context: A comparison between online and paper modes of administration
Source: Heliyon. 2022 Jun 16;8(6):e09743. doi: 10.1016/j.heliyon.2022.e09743 (PMC9218149; doi:10.1016/j.heliyon.2022.e09743)
Supplement: Questionnaire_English R1 2022 01 08 [file mmc1.docx]

**STUDENT’S ICT ATTITUDE IN LANGUAGE LEARNING**

*** Section 1. Demographic information**

**Q1. Your gender**

| a. female | b. male |
| --- | --- |

**Q2. Your year**

a. 1^st^ year b. 2^nd^ year c. 3^rd^ year d. 4^th^ year

**Q3. Your age**: …

**Q4. Your major**: …

**Section 2. Attitude towards ICT**

**Q5. Please, rate the following statements**

|  | disagree | partly disagree | partly agree | agree |
| --- | --- | --- | --- | --- |
| 1. I use a computer as part of my English learning process. | 1 | 2 | 3 | 4 |
| 2. Using a computer for English learning is very  important to me. | 1 | 2 | 3 | 4 |
| 3. Using a smartphone for English learning is very  important to me. | 1 | 2 | 3 | 4 |
| 4. Using a tablet for English learning is very important to  me. | 1 | 2 | 3 | 4 |
| 5. Using a computer for English learning makes me  happy. | 1 | 2 | 3 | 4 |
| 6. Using ICT tools for learning makes me happy. | 1 | 2 | 3 | 4 |
| 7. I use ICT tools for learning because I'm very  interested in IT. | 1 | 2 | 3 | 4 |
| 8. I save time if I use a computer for English learning. | 1 | 2 | 3 | 4 |
| 9. I can focus on English learning more if I use ICT tools. | 1 | 2 | 3 | 4 |
| 10. I can understand the English material much more  easily if I use ICT tools. | 1 | 2 | 3 | 4 |
| 11. I can remember what I have learnt better if I use  ICT tools. | 1 | 2 | 3 | 4 |
| 12. ICT tools play an important role in my English  learning process. | 1 | 2 | 3 | 4 |
| 13. ICT tools make English learning faster for me. | 1 | 2 | 3 | 4 |
| 14. ICT tools improve my English grades. | 1 | 2 | 3 | 4 |
| 15. Using ICT tools for English learning is very important  to me. | 1 | 2 | 3 | 4 |
| 16. I cannot learn without using ICT tools. | 1 | 2 | 3 | 4 |
| 17. I save time if I use ICT tools for English learning. | 1 | 2 | 3 | 4 |
| 18. Information is much more easily available by  using ICT tools than by visiting the library. | 1 | 2 | 3 | 4 |
| 19. The English material covered does not allow for the  use of ICT tools in class. | 1 | 2 | 3 | 4 |
| 20.The English material covered does not allow for the  use of ICT tools at home. | 1 | 2 | 3 | 4 |
| 21. Teachers give us guidance on how to use ICT  tools for English learning tasks to be completed at home. | 1 | 2 | 3 | 4 |
| 22. Teachers give us guidance on how to use ICT  tools for English learning in class. | 1 | 2 | 3 | 4 |
| 23. Teachers support the use of ICT tools for English  learning. | 1 | 2 | 3 | 4 |
| 24. My teachers use a computer during their English classes. | 1 | 2 | 3 | 4 |
| 25. My teachers expect me to use a computer as  part of my English learning process. | 1 | 2 | 3 | 4 |
| 26. Virtual English learning environments are used in the  courses I’m enrolled in. | 1 | 2 | 3 | 4 |
| 27. ICT tools create a better atmosphere in the English  classroom. | 1 | 2 | 3 | 4 |
| 28. Teachers should incorporate the use of ICT  tools into their English teaching. | 1 | 2 | 3 | 4 |
